# Effects of Arachidonic Acid Supplementation on Acute Anabolic Signaling and Chronic Functional Performance and Body Composition Adaptations

**DOI:** 10.1371/journal.pone.0155153

**Published:** 2016-05-16

**Authors:** Eduardo O. De Souza, Ryan P. Lowery, Jacob M. Wilson, Matthew H. Sharp, Christopher Brooks Mobley, Carlton D. Fox, Hector L. Lopez, Kevin A. Shields, Jacob T. Rauch, James C. Healy, Richard M. Thompson, Jacob A. Ormes, Jordan M. Joy, Michael D. Roberts

**Affiliations:** 1 Department of Health Sciences and Human Performance, The University of Tampa, Tampa, FL, United States of America; 2 Molecular and Applied Sciences Laboratory, School of Kinesiology, Auburn University, Auburn, AL, United States of America; 3 The Center for Applied Health Sciences, 4302 Allen Road, STE 120, Stow, OH, 44224, United States of America; University of Birmingham, UNITED KINGDOM

## Abstract

**Background:**

The primary purpose of this investigation was to examine the effects of arachidonic acid (ARA) supplementation on functional performance and body composition in trained males. In addition, we performed a secondary study looking at molecular responses of ARA supplementation following an acute exercise bout in rodents.

**Methods:**

Thirty strength-trained males (age: 20.4 ± 2.1 yrs) were randomly divided into two groups: ARA or placebo (i.e. CTL). Then, both groups underwent an 8-week, 3-day per week, non-periodized training protocol. Quadriceps muscle thickness, whole-body composition scan (DEXA), muscle strength, and power were assessed at baseline and post-test. In the rodent model, male Wistar rats (~250 g, ~8 weeks old) were pre-fed with either ARA or water (CTL) for 8 days and were fed the final dose of ARA prior to being acutely strength trained via electrical stimulation on unilateral plantar flexions. A mixed muscle sample was removed from the exercised and non-exercised leg 3 hours post-exercise.

**Results:**

Lean body mass (2.9%, p<0.0005), upper-body strength (8.7%, p<0.0001), and peak power (12.7%, p<0.0001) increased only in the ARA group. For the animal trial, GSK-β (Ser9) phosphorylation (p<0.001) independent of exercise and AMPK phosphorylation after exercise (p-AMPK less in ARA, p = 0.041) were different in ARA-fed versus CTL rats.

**Conclusions:**

Our findings suggest that ARA supplementation can positively augment strength-training induced adaptations in resistance-trained males. However, chronic studies at the molecular level are required to further elucidate how ARA combined with strength training affect muscle adaptation.

## Introduction

Fatty acids supplementation has received a high degree of popularity for increasing health benefits. For instance, eicosapentaenoic acid (EPA) and docosahexaenoic acid (DHA) supplementation have been utilized to reduce skeletal muscle inflammation and protein breakdown, as well as neural and cardiometabolic health [1, 2]. Specifically, one such fatty acid that has garnered a progressive amount of scrutiny over recent years is arachidonic acid (ARA). ARA is a long-chain polyunsaturated fatty acid (20:4n-6) that exists in relatively low amounts in the typical American diet [3]. In this regard, ARA is primarily consumed through meat and fish products. In the human body, ARA resides in the phospholipid bi-layer of cell membranes at concentrations contingent upon dietary intake [4]. While the literature illustrates the responsiveness of cell membrane composition to dietary intakes, phospholipids also appear to be dependent upon activity level suggesting increased ARA turnover or demand. For example, Andersson et al. (2000) noted a lower n-6:n-3 ratio and lower total n-6 fatty acids in phospholipids of exercising individuals [5, 6]. Likewise, Helge et al. (2001) similarly demonstrated that a lower n-6:n-3 ratio exists in strength-trained individuals [7].

Furthermore, ARA drives the inflammatory response to strength training [7]. To this end, this inflammatory response appears to be mediated by ARA liberated from plasma membranes via phospholipase A2 (PLA_2_). The free ARA follows its metabolic fate to generate bioactive lipid mediators known as eicosanoids by one of three biochemical pathways involving lipoxygenases (LOX), P450 epoxygenases or cyclooxygenases (COX) [8]. COX enzyme plays an important role for converting ARA to form postranoids such as Prostaglandins [9–11]. In addition, Prostaglandin E_2_ (PGE_2_) and Prostaglandin F_2_-α (PGF_2_-α) appear to be associated with protein degradation and synthesis in skeletal muscle, respectively [12]. Moreover, PGF_2_-α has been shown to elicit essential pathways responsible for myogenic proliferation, differentiation, and fusion *in vitro* [13, 14]. For instance, previous research demonstrated that *in vitro* ARA supplementation stimulates prostaglandins release and skeletal muscle hypertrophy via a COX-2 dependent pathway [9]. Moreover, animal model studies also demonstrated that COX-inhibitors consumption attenuates muscle hypertrophy and regrowth from muscle atrophy [15].

However, in humans, after COX-inhibitors consumption, ARA-derived prostaglandins have demonstrated conflicting results concerning their role in acute post-strength training muscle protein synthesis and training-induced adaptations [16, 17]. For instance, previous research examined the effects of COX-inhibitors (administered as Non-steroidal anti-inflammatory drugs [NSAIDs] Ibuprofen or acetaminophen) on muscle protein synthesis responses after a high intensity eccentric exercise bout (e.g. 120% of the concentric maximum) in young males (˜26 years old). The authors demonstrated that COX inhibitors, which prevent ARA-derived prostaglandins formation, completely attenuate muscle protein synthesis 24 hours following eccentric exercise [18]. In addition, PGF_2_-α, which is a COX product and has been show to stimulate protein synthesis significantly, increased only in PLA when compared to NSAIDs conditions. Furthermore, the same group investigated the effects of daily consumption of COX-inhibitors (acetaminophen or ibuprofen) during 12 weeks of strength training in older adults. The authors hypothesized that experimental groups would demonstrate lower muscle mass and strength benefits than placebo. Inquiringly, contrary to the authors’ hypothesis, both experimental groups demonstrated greater muscle strength and volume increases than placebo. In addition, COX-1 and COX-2 proteins expression significantly increased from baseline across the three groups. The aforementioned results suggest that the role of ARA in muscle hypertrophic adaptations is equivocal. Still, sparse evidence has examined whether additional supplementation with ARA yields significant benefits to functional performance and body composition adaptations in strength-trained individuals. Research conducted by Roberts et al. in 2007 investigated the effects of ARA supplementation (1g•d^-1^ vs. placebo) in strength-trained subjects for eight weeks [19]. While there was a significant increase in anaerobic peak power, there did not appear to be any improvements between ARA and a placebo supplement in body composition or strength measures. Moreover, Roberts et al. (2007) co-administered 90g•d^-1^ of supplemental protein to the ARA and placebo groups, which may have negated the potential benefit of ARA.

Taken together the above-mentioned outcomes, it seems plausible to say that the possible mechanism in which ARA drive skeletal muscle adaptations to strength training and the effects of ARA supplementation on body composition and functional performance in strength-trained populations have not been elucidated. Therefore, the purpose of this study was two-fold: Phase 1) to examine the effects of 1.5g•d^-1^ of ARA supplementation on body composition, muscle strength, and anaerobic power in strength-trained individuals participating in non-periodized strength-training; and Phase 2) to examine if the effects of electrical stimulation after 8-days of ARA supplementation in rats enhance post-exercise anabolic signaling mechanisms and muscle protein synthesis. We hypothesized that ARA supplementation can increase the post-exercise anabolic signaling in rat skeletal muscle, and that younger strength-trained individuals consuming ARA supplementation will demonstrate greater functional performance and body composition adaptations.

## Methods

### Experimental Design

The current study was designed to investigate the molecular, functional performance and body composition adaptations induced by chronic (e.g. 8-wk of ARA supplementation combined with strength training) and semi-chronic supplementation (e.g. eight days of ARA supplementation prior to the electrical stimulation simulating strength training) in a randomized, double-blind, placebo-controlled, parallel design. In order to address the effects of semi-chronic ARA supplementation on molecular adaptations related to protein synthesis and breakdown signaling, we used a rat model in which one stimulated exercise bout was delivered to the gastrocnemius muscle following 8-days of ARA supplementation whereby the dosage was approximately equal to the human dosage used (e.g. 1.5g•d^-1^). The exercise model was selected to mimic a strength-training stimulus based on its efficacy in inducing acute increases in protein synthesis and to promote skeletal muscle hypertrophy in a chronic fashion [20, 21]. The human model was used to investigate the chronic effects of ARA supplementation combined with strength training on functional performance and body composition adaptations followed by an 8-wk, non-periodized, hypertrophy-oriented regimen that was performed three times a week. Briefly, prior to beginning of training, familiarization sessions and baseline measures were performed. In sequential order, baseline assessments included: body composition, muscle thickness of the vastus lateralis, 1-repetition maximum (1RM) leg press, 1RM bench press, and lower-body peak power output using the cycle ergometer Wingate test. Subsequent to baseline testing, participants were classified into quartiles according to their lean body mass (LBM). Then, participants from each quartile were randomly assigned to either the supplement + strength training (Ex ARA; n = 15) or placebo + strength training (Ex CTL; n = 15) group. The following week, participants began training and supplementation. Participants underwent the training program for 8 weeks. Upon completion of week 8, participants returned to the laboratory ~48h after their last training session in order to collect post-training assessments. Participants were instructed to maintain their typical dietary habits throughout the study period. In this current study, the human and animal models were used for different purposes; the animal model was selected to investigate the mechanisms underlying acute responses related to anabolic and catabolic signaling induced by ARA supplementation, while the human model was used to elucidate the strength training-induced adaptations combined with ARA supplementation.

### Phase 1 –Human Trial

#### Participants

Participants included thirty strength-trained males (mean ± SD; age = 20.4 ± 2.1 years; height = 177.7 ± 6.3 cm; body mass = 76.4 ± 7.8 kg and LBM: 57.7 ± 5.1 kg). Participants were excluded from participation if they were currently taking any medications, anti-inflammatory drugs, or dietary supplements that could influence athletic performance. No medical disorders, diseases, or musculoskeletal injuries were reported among participants. Additionally, participants were required to possess a minimum strength training age of 2 years. All participants read and signed an informed consent approved by the University of Tampa Institutional Review Board (i.e. review-form 13–50) in accordance with the Declaration of Helsinki as well as a health history questionnaire.

#### Supplementation

Each participant consumed two soft gels (1.5g ARASYN; 40% arachidonic acid) of either X-Factor (Molecular Nutrition, Jupiter, FLA, USA) or a visually identical placebo (i.e. corn oil) once per day approximately 45 minutes prior to the training session. Empty supplement bottles were returned to investigators to ensure compliance before the commencement of all training sessions. Supplement compliance was high (i.e., 99.4%)

#### Body Composition

A whole body scan was performed using a Lunar Prodigy Dual X-ray absorptiometry (DEXA) apparatus (Hologic, Bedford, MA, USA) to measure body composition. Lean body mass (LBM) and fat mass (FM) were determined for the total body with the subject lying in a supine position with the knee extended and instructed not to move for the entire duration of the scan, which took approximately 10-min. Results from each scan were uploaded and accessed on computer that was directly linked to the DEXA device. Additionally, ultrasonography (GE LOGIQ e; General Electric Company, Fairfield, CT, USA) was used to determine the thickness of the vastus lateralis muscle using an electronic linear array probe with a wave frequency of 8.0 MHz. Muscle thickness was assessed midway between the greater trochanter of the femur and lateral epicondyle of the knee and it was defined as the distance between the interface of the muscle tissue and sub-cutaneous fat to the bone (femur). This specific spot (e.g. midway distance between the greater trochanter and lateral epicondyle) was marked with a permanent marker during baseline measures and participants were instructed to keep their mark throughout the duration of the study in order to maintain consistency of the site of measurement. To obtain the images, subjects laid supine with their legs fully extended and their muscles relaxed. A water-soluble gel was applied to the transducer to aid acoustic coupling and remove the need to contact the skin; this eliminated deformations of the muscle that can occur when pressure is directly applied to the skin and underlying muscle. Scans were performed on the right leg with the transducer-oriented perpendicular to the vastus lateralis. The same investigator performed ultrasound assessments pre-to-post experimental period and was blinded to the treatment groups. Body composition and muscle thickness measures were acquired at weeks 0 and 8. The coefficient of variation (CV) for body composition and muscle thickness assessments were 1.5% and 2%, respectively.

#### Muscle Strength and Power

Muscle strength was assessed through 1RM leg press and 1RM bench press. A trained tester that was certified by the National Strength and Conditioning Association (NSCA-CSCS-certified) observed strength testing and loads were increased incrementally until maximal load or failure at a given load was reached. In brief, participants performed a general warm-up and a specific warm-up consisting of two sets. During the first set, participants performed 10 repetitions with 50% of the predicted 1RM. In the second set, they performed five repetitions with 75% of the predicted 1RM. After the second warm-up set, participants rested for 3-minutes. Then, each participant had up to five attempts to achieve the 1RM load. Strong verbal encouragement was given throughout the 1RM test. Muscle power was assessed during maximal Wingate cycle ergometer sprinting. During the cycling test, the participant was instructed to cycle against a predetermined resistance (7.5% of body mass) as fast as possible for 10 seconds. The saddle height was adjusted for each individual in order to produce a 5–10° knee flexion while the foot was in the low position of the central void. A standardized verbal stimulus was provided to the participant. Power output was recorded in real time by a computer connected to the Monark standard cycle ergometer (Monark^®^ model 894e, Vansbro, Sweden) during a 10-second sprint test. Wingate peak power (PP) was recorded using Monark Anaerobic test software (Monark Anaerobic Wingate Software, Version 1.0). Muscle strength and power were acquired at weeks 0 and 8. The CV for 1RM testing and PP were 3.4% and 4.0%, respectively.

#### Strength Training Regimen

The participants underwent a non-periodized, hypertrophy-oriented, whole body regimen for 8 weeks that consisted primarily of free-weight, compound movements. Training occurred three days per week (Table 1). Exercises and repetition schemes remained the same for all 8 weeks. Training loads for a given exercise were increased if the subject could lift a given weight for 4 sets of 8–12 repetitions with proper technique prior to muscular failure (i.e. 4 x 8–12RM). A 2-min rest interval was allowed between sets while 3 minutes were respected between exercises. Repetitions were performed with a 3:1 concentric to eccentric contraction cadence. Research assistants and a trained Certified Strength and Conditioning Specialist (CSCS) monitored subjects to ensure training compliance, proper tracking of lifting volume, and proper exercise technique was executed.

**Table 1:** Schematic of 8-week resistance training regimen in humans.

Monday–Lower Body	Wednesday–Back, Biceps	Friday–Chest, Shoulders, Triceps
Leg Press	Bent Over Rows	Bench Press
Leg Extension	Lat Pull-down	Military Press
Leg Curls	Barbell Curls	Skull Crunchers
Hyperextensions		Barbell Shrugs

### Phase 2—Animal Trial

#### Feeding and Acute Strength Exercise Stimulation Bout

The Auburn University Institutional Review Board approved all experimental procedures described herein (i.e. Animal subjects review form: 2014–2481). Male Wistar rats (~250 g, approximately 8 weeks old) were purchased from Harlan Laboratories (Indianapolis, IN, USA) and were allowed to acclimate in the animal quarters for 5 days prior to experimentation. Briefly, animal quarters were maintained on a 12 h light: 12 h dark cycle, at ambient room temperature, and water and standard rodent chow (18.6% protein, 44.2% carbohydrate, 6.2% fat; Teklad Global #2018 Diet, Harlan Laboratories) were provided to animals ad libitum.

Eight days prior to the acute strength training experiment, rats were gavage-fed once daily with either 1.2 ml of tap water (CTL) or 44 mg ARA (Molecular Nutrition) dissolved in ~1 ml of tap water. This feeding paradigm was meant to pre-load the animals with ARA prior to one acute exercise bout. Feeding took place under light isoflurane anesthesia in order to reduce the daily repetitive stress of gavage feeding as previously described [22]. This dose of ARA approximately equaled a human dosage used in the current study (i.e., dose per the species conversion calculations of Reagan-Shaw et al. (2008) [23] whereby the human body mass for an average male was assumed to be 80 kg.

The morning of the acute strength training experiment (day 8 or 9), food was removed from home cages resulting in a ~5–6 h fast. Rats were then transported to the Molecular and Applied Sciences Laboratory and were allowed to acclimate for approximately 1–2 hours. Thereafter, rats were administered a final dose of either 1.2 ml of tap water (CTL, n = 10) or 44 mg ARA (n = 11) dissolved in ~1 ml of tap water via gavage feeding under light isoflurane anesthesia. Rats then remained under isoflurane anesthesia for an electrically-stimulated lower body unilateral plantar flexion exercise per the modified methods of Baar and Esser [21].

Briefly, animals were fastened to an apparatus to allow the two hind limbs to move freely. Two subcutaneous electrodes connected to a Grass S48 Stimulator (Grass Medical Instruments, Quincy, MS, USA) were placed parallel to the gastrocnemius in each rat’s right leg. Four sets of 8 stimulations then occurred with the following settings: 70 mV, 100 Hz, 2,000 ms train duration, 0.2 TPS train rat, and 0.2 ms duration. Between sets rats were allowed 2 min of recovery. Following the electrically-stimulated exercise bout, rats were allowed to recover 180 minutes prior to being euthanized under CO2 gas. Rats were injected with puromycin dihydrochloride 30 min prior to euthanasia (5.44 mg in 1 ml of diluted in phosphate buffered saline; Ameresco) as a metabolic tracer in order to determine skeletal muscle protein synthesis (MPS) via the SUrface SEnsing of Translation (SUnSET) method described in detail elsewhere [24]. Hence, this protocol allowed us to analyze the 3-h post-exercise anabolic response in animals that were supplemented 8 days prior to the acute stimulation bout.

#### Skeletal Muscle Processing

Immediately following euthanasia two 50 mg pieces of mixed gastrocnemius muscle were harvested using standard dissection techniques and placed in homogenizing buffer [Tris base; pH 8.0, NaCl, NP-40], sodium deoxycholate, SDS with added protease and phosphatase inhibitors (G Biosciences, St. Louis, MO, USA)] and Ribozol (Ameresco, Solon, OH, USA) for immunoblotting and mRNA analyses, respectively. Muscle samples placed in Tris base homogenizing buffer were homogenized using a 1.7 ml tube using a tight-fitting micropestle, insoluble proteins were removed with centrifugation at 500xg for 5 min at 4°C, and supernatants were assayed for total protein content using a BCA Protein Assay Kit (Thermo Scientific, Waltham, MA, USA) prior to immunoblotting sample preparation. Muscle, samples placed in Ribozol were subjected to total RNA isolation according to manufacturer’s instructions, and concentrations were performed using a NanoDrop Lite (Thermo Scientific) prior to cDNA synthesis for mRNA analyses. Extra gastrocnemius muscle not processed during dissections was flash-frozen in liquid nitrogen and stored at -80°C for later potential analyses.

#### Directed Akt-mTOR Phosphoproteomics

The PathScan^®^ Akt Signaling Antibody Array Kit (Chemiluminescent Readout; Cell Signaling, Danvers, MA, USA) containing glass slides spotted with antibodies was utilized to detect phosphorylated proteins predominantly belonging to the Akt-mTOR signaling network [p-Akt (Ser473), p-rps6 (Ser235/236), p-AMPK-α (Thr172), p-GSK-3α (Ser21), p-GSK-3β (Ser9), p-p70s6k (Thr389), p-4E-BP1 (Thr37/46)]. Briefly, gastrocnemius homogenates were diluted to 1.0 μg/μl using cell lysis buffer provided by the kit and assayed according to manufacturer’s instructions. Slides were developed using an enhanced chemiluminescent reagent provided by the kit, and spot densitometry was performed through the use of a UVP Imager and associated densitometry software (UVP, LLC, Upland, CA, USA). Although each sample included a positive internal control, the densities of these controls were relatively variable. Therefore, sample-sample normalization was instead performed by taking the summated density of all of the phosphorylated targets. The calculation of each phosphorylated target was as follows:

(Density value of the phosphorylated protein – negative control) / summated density of all of the phosphorylated targets

#### SUnSET Method For MPS Determination

As mentioned previously, the SUnSET method was used in order to examine if different dietary treatments with or without stimulated strength exercise differentially affected MPS. In brief, the SUnSET technique involves the use of an anti-puromycin antibody for the immunological detection of puromycin-labelled peptides (i.e. metabolic tracer). Initially, developed for use in cultured cells, SUnSET allows for the detection in protein synthesis changes in whole cell lysates using western blotting. Thence, 2 μg/μl gastrocnemius Western blotting preps were made using 4x Laemmli buffer. Thereafter, 30 μl of prepped samples were loaded onto 12% SDS-polyacrylamide gels and subjected to electrophoresis (200 V @ 75 min). Proteins were then transferred to polyvinylidene difluoride membranes (Whatman^™^, Westran^®^ Clear Signal), and membranes were blocked for 1 h at room temperature with 5% nonfat milk powder. Mouse anti-puromycin IgG (1:5,000; Millipore) was incubated with membranes overnight at 4°C in 5% bovine serum albumin (BSA), and the following day membranes were incubated with anti-mouse IgG secondary antibodies (1:2,000, Cell Signaling) at room temperature for 1 h prior to membrane development. Membrane development was performed using an enhanced chemiluminescent reagent (Amersham, Pittsburgh, PA, USA), and band densitometry was performed using a UVP Imager and associated densitometry software (UVP, LLC, Upland, CA, USA). Given that mixed gastrocnemius muscle yields varying normalizer protein levels (e.g., beta-actin and GAPDH; *unpublished observations by MDR*), membranes were incubated with Coomassie stain in order to visually verify equal protein loading between lanes.

#### Western Blotting

For determination of gastrocnemius phospho-eEF2 (Thr 56) protein levels 2 μg/μl gastrocnemius Western blotting preps were made using 4x Laemmli buffer. Thereafter, 30 μl of prepped samples were loaded onto 12% SDS-polyacrylamide gels and subjected to electrophoresis (200 V @ 75 min). Proteins were then transferred to polyvinylidene difluoride membranes (Whatman^™^, Westran^®^ Clear Signal), and membranes were blocked for 1 h at room temperature with 5% nonfat milk powder.

Rabbit anti-phospho-eEF2 (Thr 56) IgG (1:1,000; Cell Signaling) was incubated with membranes overnight at 4°C in 5% bovine serum albumin (BSA), and the following day membranes were incubated with anti-rabbit or anti-mouse IgG secondary antibodies (1:2,000, Cell Signaling), respectively, at room temperature for 1 h prior to membrane development. Membrane development was performed as described above. As described above, membranes were stained with Coomassie in order to visually ensure between-lane protein loading equality.

#### Real-Time RT-PCR

Total RNA (1 μg) was reverse transcribed into cDNA for real time PCR analyses using a commercial cDNA synthesis kit (Quanta Biosciences, Gaithersburg, MD, USA). Real-time PCR was performed using SYBR-green-based methods with gene-specific primers [FP receptor (PTGFR), EP3 receptor (PTER3) prostaglandin F synthase (PGFS), prostaglandin E synthase (PTGES), prostaglandin E synthase 2 (PTGES2), prostaglandin E synthase 3 (PTGES3), cytosolic phospholipase 2 (cPLA2), cyclooxygenase-2 (COX-2), Atrogin-1, MuRF-1, interleukin-6 (IL-6), androgen receptor (AR), myogenic differentiation 1 (MyoD), myogenin, paired box 7 (Pax7), beta-2 microglobulin (B2M; normalizer)] designed using primer designer software (Primer3Plus, Cambridge, MA, USA). The forward and reverse primer sequences are presented in Table 2.

**Table 2:** Sequence of primers used in real-time polymerase chain reaction.

Genes	Forward	Reverse
**AR**	CGCTTCTACCAGCTCACCAA	TCAGGAAAGTCCACGCTCAC
**Atrogin-1**	CTACGATGTTGCAGCCAAGA	GGCAGTCGAGAAGTCCAGTC
**B2M**	CCCAAAGAGACAGTGGGTGT	CCCTACTCCCCTCAGTTTCC
**COX-2**	AAAGCCTCGTCCAGATGCTA	ATGGTGGCTGTCTTGGTAGG
**cPLA2**	TTAACCTGCCGTATCCCTTG	CTTCAATCCTTCCCGATCAA
**IL-6**	ATCTGCCCTTCAGGAACAGC	GAAGTAGGGAAGGCAGTGGC
**MuRF-1**	AGTCGCAGTTTCGAAGCAAT	AACGACCTCCAGACATGGAC
**MyoD**	GAGTGGCCAGGACCTCTTTC	AACAGGGATGTGGAAGGCA
**Myogenin**	TCCCAGATGAAACCATGCCC	GTCTGACACCAACTCAGGGG
**Pax7**	TCCATCTCAGCCAGTTGCAG	CAAGCTGTCTCCTGGCTTGA
**PGFS**	CTAAGATGGCAGCCCTAGCC	GCCTCTGAGAGTCGAGCATC
**PTER3**	AATGCGCTCAGTCCTCTGTT	CCTTTACGTTCCTCCAACGA
**PTGES**	GGGCTAAGGATGAGGGCTTC	CCCTGAGACACACACCAGTC
**PTGES2**	TCTGGAAGCCTTTGACGACC	ACCAAGGCTGGATGTGTGAG
**PTGES3**	ACTTGCACTGTCAGTATGGCA	GGTTTTCCAGCCAGGGCATA
**PTGFR**	CCAGGAGTTGGGATCACTGT	ACCGTAGCCACTGATGGAAC

Primer efficiency curves for all genes were generated and efficiencies ranged between 90% and 110%, and melt curve analyses demonstrated that one PCR product was amplified per reaction.

#### Statistical Analysis

Visual inspection of boxplots and Shapiro-Wilk testing confirmed that dependent variables were normally distributed. For the human trial, a two-way ANOVA with repeated measures was performed assuming time (baseline and post) and group (Ex CTL and Ex ARA) as fixed factors. Whenever a significant F-value was obtained, a post-hoc with Tukey´s adjustment was performed for multiple comparisons. In addition, we presented the mean, upper and lower limits values of confidence intervals of the absolute differences (CI_diff_) as this approach allows us to know how much the experimental groups affected the dependent variables investigated, rather than only the level of statistical significance. In this regard, the confidence interval includes the value range in which the true population mean of the difference is likely to be. Positive and negative confidence intervals that did not cross zero were considered significant. For the animal trial, a two-way ANOVA was performed assuming limb (exercised and non-exercised) and group (Ex CTL and Ex ARA) as fixed factors. Because there were only two treatment legs and conditions, significant main effects or interactions were followed with dependent and independent samples t-tests, respectively. The significance levels was previously set at p<0.05. All data are presented as means ± standard errors.

## Results

### Phase 1 –Human Trial

#### Body Composition and Muscle Thickness

**Fig 1:**
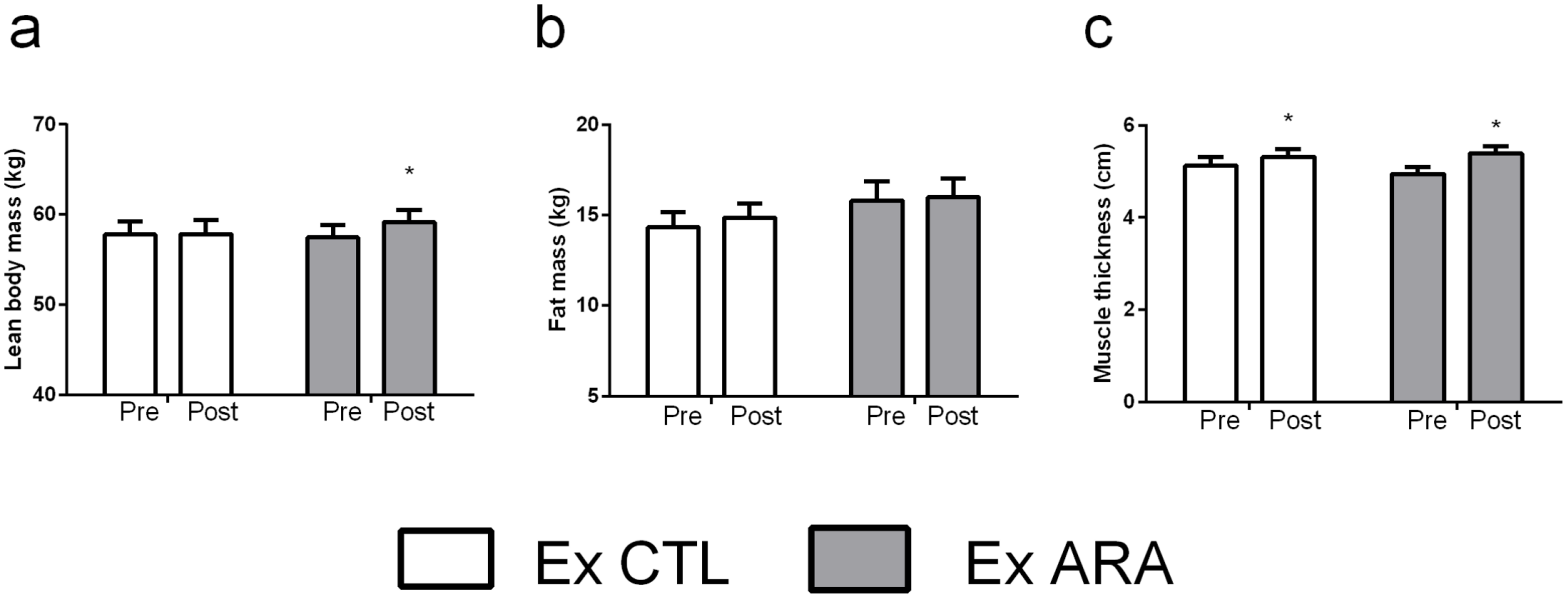
Pre- and post-testing values for lean body mass (a), fat mass (b), and muscle thickness (c) for Ex CTL and Ex ARA groups. *—Indicates p <0.05 for within-group comparisons.

Body composition and muscle thickness assessments are presented in Fig 1. No significant differences between groups in LBM and FM were detected at the baseline (p>0.05). There was a significant group by time interaction for LBM (p<0.0005). Post-hoc comparisons revealed that only the Ex ARA group increased LBM significantly following 8 weeks of supplementation (2.9%, p<0.0007) (Fig 1a). In addition, Ex ARA group demonstrated a CI_diff_ not crossing zero for the increase in LBM (Ex ARA: 95% CI_diff:_ mean 1.6kg, lower limit 0.7, upper limit 2.6kg; Ex CTL: 95% CI_diff:_ mean 0.04kg, lower limit -0.9, upper limit 0.99kg). There were no pre-to-post significant differences in FM for Ex ARA and Ex CTL groups (p>0.05) (Fig 1b). However, it is important to highlight that Ex CTL demonstrated CI_diff_ not crossing zero for the increase in FM (Ex CTL: 95% CI_diff:_ mean 0.5kg, lower limit 0.10, upper limit 0.9kg; Ex ARA: 95% CI_diff:_ mean -0.17kg, lower limit -0.2, upper limit 0.6kg). For muscle thickness, no significant differences between groups were detected at baseline (p>0.05). There was a significant main effect for time (p<0.0001) in which muscle thickness increased significantly in the Ex ARA and Ex CTL groups (9.0%, p<0.0001 and 3.7%, p<0.01), respectively. Furthermore, both groups demonstrated CI_diff_ not crossing zero for the increase in muscle thickness (Ex CTL: 95% CI_diff:_ mean 0.19cm, lower limit -0.03, upper limit 0.34cm; Ex ARA: 95% CI_diff:_ mean 0.4cm, lower limit -0.2, upper limit 0.6cm).

#### Performance Assessments

**Fig 2:**
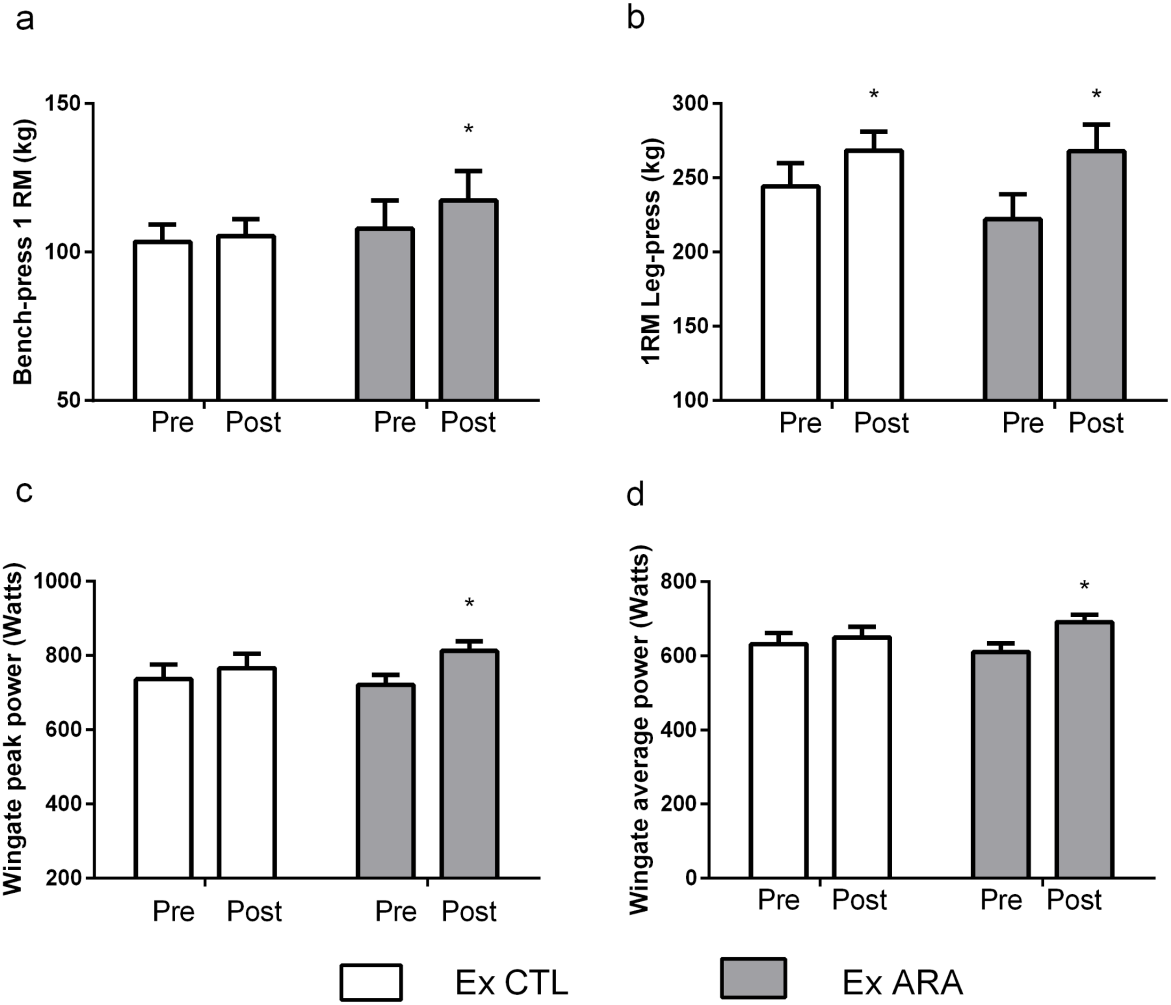
Pre- and post-testing values for bench press 1RM (a), leg press 1RM (b), Wingate peak power (c), and Wingate average power (d for Ex CTL and Ex ARA groups. *—Indicates p <0.05 for within-group comparisons.

All performance variables are presented in Fig 2. No significant differences between-groups in maximum dynamic strength were detected at the baseline for all 1RM tests (p>0.05). There was a significant group by time interaction for upper-body strength (p<0.0001). Post-hoc comparisons revealed that only the Ex ARA group significantly increased 1RM bench-press (8.7%, p<0.0001) (Fig 2a). In addition, only Ex ARA demonstrated CI_diff_ not crossing zero for the pre-post differences (95% CI_diff:_ mean 9.5kg, lower limit 6.2, upper limit 12.7kg).

For lower-body strength, there was a significant main effect for time (p<0.0001) in which 1RM leg-press increased significantly in the Ex ARA and Ex CTL groups (20.5%, p<0.0001 and 9.9%, p<0.002) (Fig 2b), respectively. However, it is important highlight that none of the experimental groups demonstrated significant CI_diff_ (Ex CTL: 95% CI_diff:_ mean 24.2kg, lower limit -27.8, upper limit 76.3kg; Ex ARA: 95% CI_diff:_ mean 45.7kg, lower limit -6.3, upper limit 97.8kg). Furthermore, both the Ex ARA and Ex CTL groups significantly increased total-body strength (14.4%, p<0.0001 and 7.5%, p<0.01), respectively. In addition, for total-body strength (i.e. Ʃ 1RM leg-press and 1RM bench press values), only Ex ARA demonstrated a significant CI_diff_ (Ex CTL: 95% CI_diff:_ mean 7.6kg, lower limit -8.1, upper limit 23.5kg; Ex ARA: 95% CI_diff:_ mean 54.5kg, lower limit 38.6, upper limit 70.4kg).

No significant differences between-groups in PP and average power assessments were detected at the baseline (p>0.05). There were significant group by time interactions for PP (p<0.02) and average power (p<0.006). Subsequent post-hoc analysis revealed that only the Ex ARA group increased significantly the PP (12.7%, p<0.0001), (Fig 2c) and average power (13.2%, p<0.0001), (Fig 2d). Furthermore, only Ex ARA demonstrated a significant CI_diff_ (Ex CTL: 95% CI_diff:_ mean 17.6watts, lower limit -17.9, upper limit 53.2watts; Ex ARA: 95% CI_diff:_ mean 80.6watts, lower limit 45.0, upper limit 116.3watts).

### Phase 2: Animal Trial

#### Protein Synthesis and Anabolic Signaling

**Fig 3:**
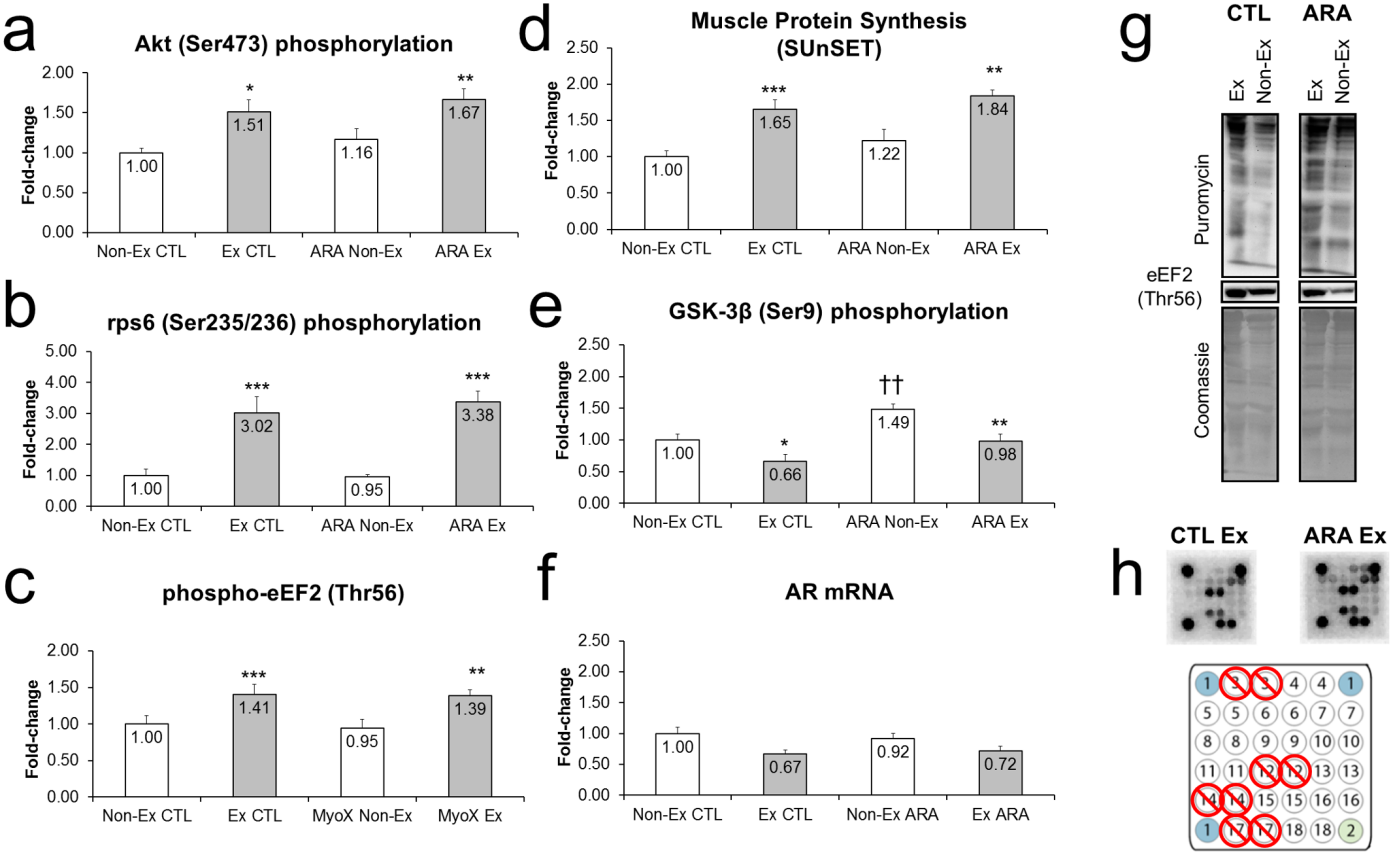
Fold-change values for Akt (Ser473) phosphorylation (a), rps6 (Ser235/236) phosphorylation (b), eF2 (Thr56) phosphorylation (c), and AR mRNA expression (f) in non-exercised (open bars) and exercised (closed bars) rodents with (right) or without arachidonic acid supplementation. Representative digital images for MPS levels and eF2 phosphorylation levels, as well as Coomassie images to verify equal protein between lands (panel g). Note that some targets were not included in the analyses due to poor and/or inconsistent signal (panel h).

The responses in protein synthesis and anabolic signaling markers following 8-days of ARA supplementation and acute exercise are presented in Fig 3. There was a significant main effect for exercise regarding increases in Akt (Ser473) phosphorylation (p<0.001) Fig 3a, rps6 (Ser235/236) phosphorylation p<0.001) Fig 3b, eEF2 (Thr56) phosphorylation (p<0.001), Fig 3c, and muscle protein synthesis (p<0.001), Fig 3d.

There was a significant main effect for group regarding phosphorylation of GSK-3β and the phosphorylation of this substrate was greater in the ARA versus the CTL (p<0.001) (Fig 3e). Moreover, basal phosphorylation of GSK-3β was greater in ARA vs CTL (p<0.01). Exercise decreased phosphorylation of GSK-3β in both ARA (p<0.01) and CTL conditions (p<0.01). Of note, there were no significant main effects for p70s6k (Thr389) or 4EBP-1 (Thr37/46) phosphorylation. Finally, there was a significant main effect for exercise for AR mRNA which demonstrated an overall decrease in AR (p = 0.015) (Fig 3f). There were no within-group differences in the CTL and ARA groups when comparing Non-Ex to Ex muscles (p>0.05). However, the exercise control group tended to decrease AR versus Non-Ex CTL (p = 0.056). This effect was not evident in the ARA group (p = 0.14).

#### Catabolic Signaling

**Fig 4:**
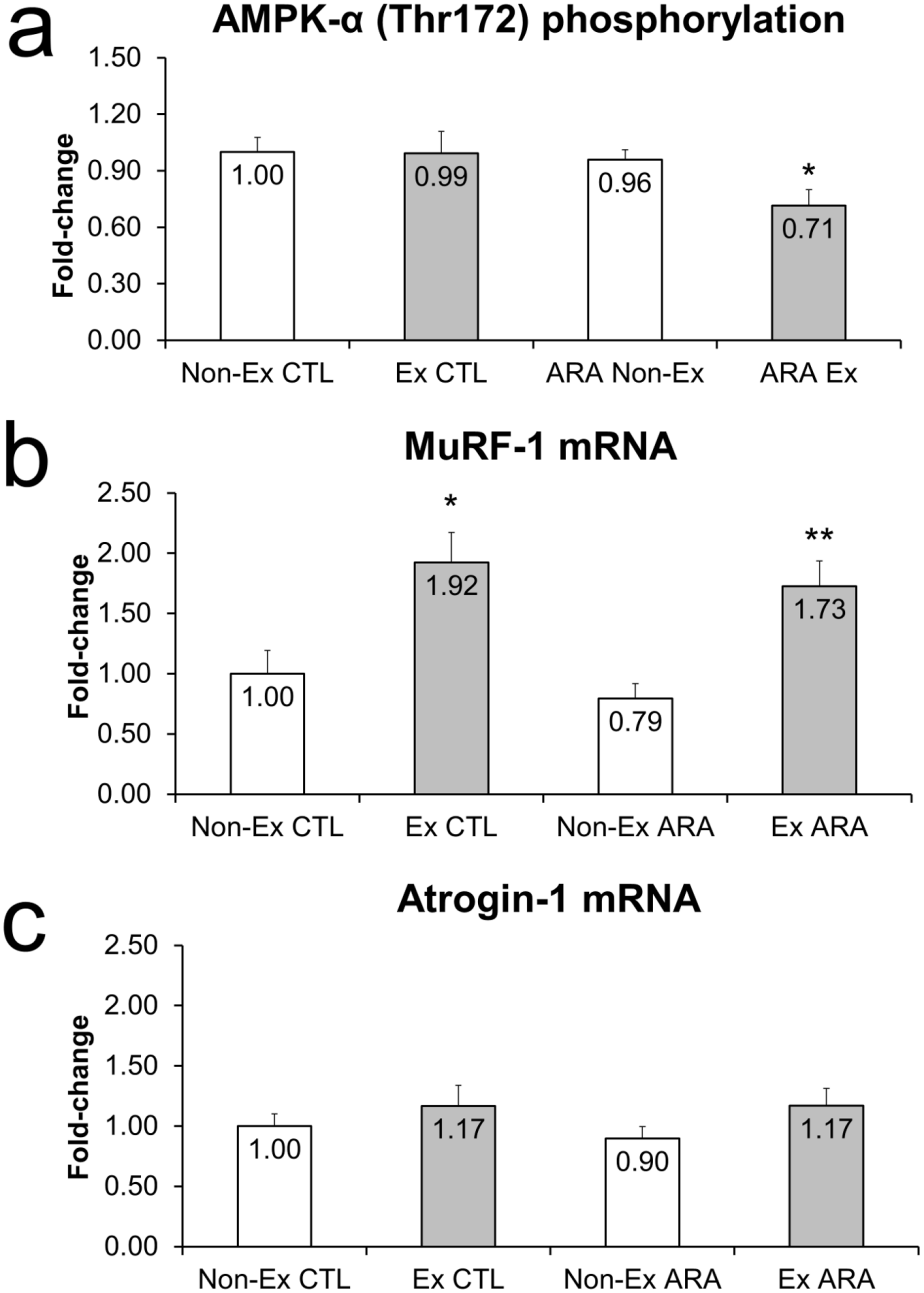
Fold-change values for AMPK-α (Thr172) phosphorylation (a), MuRF-1 mRNA expression (b), and Atrogin-1 mRNA expression (c) in non-exercised (open bars) and exercised (closed bars) rodents with (right) and without arachidonic acid supplementation.

The catabolic signaling markers following 8-days of ARA supplementation and acute exercise are presented in Fig 4. There was a significant main effect for group for AMPK phosphorylation (p-AMPK less in ARA, p = 0.041) in which phosphorylation of AMPK only decreased in the exercising ARA, but not control condition (Fig 4a). There was a significant main effect for exercise for MURF-1 mRNA (increased, p<0.001) which increased in both the control (p<0.05) and ARA conditions (p<0.01), with no differences between conditions (Fig 4b). There were no significant main effects for atrogin-1 (Fig 4c).

#### Changes in ARA-related mRNA Expression

**Fig 5:**
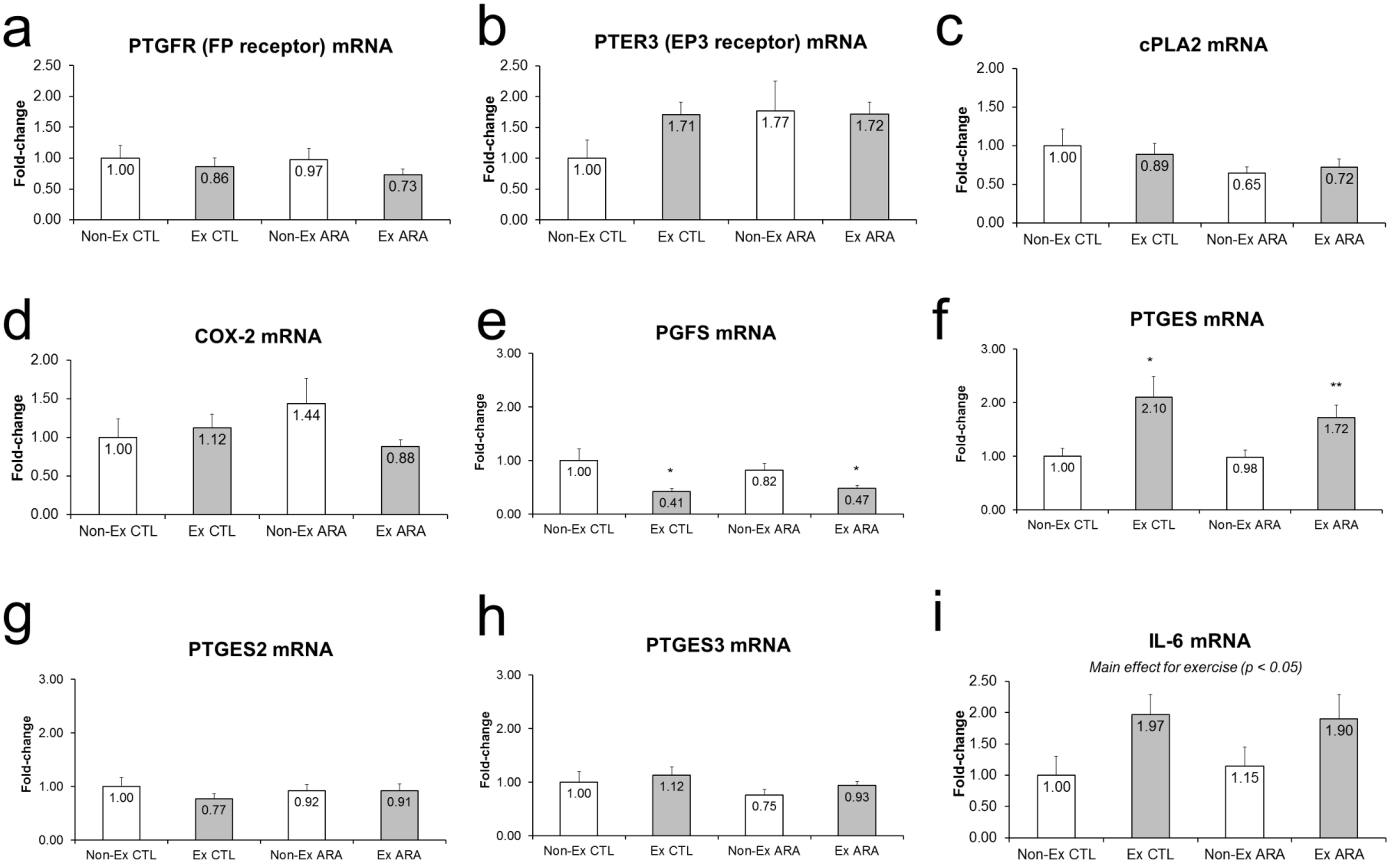
Fold-change values for PTGFR (FP receptor) mRNA expression (a), PTER3 (EP3 receptor) mRNA expression (b), cPLA2 mRNA expression (c), COX-2 mRNA expression (d), PGFS mRNA expression (e), PGTES mRNA expression (f), PGTES2 mRNA expression (g), PGTES3 mRNA expression (h) and IL-6 mRNA expression (i) in non-exercised (open bars) and exercised (closed bars) rodents with (right) and without arachidonic acid supplementation.

The responses in ARA-related mRNA expression following 8-days of ARA supplementation and acute exercise are presented in Fig 5. There were no significant main effects for PTGFR (FP receptor) mRNA, PTER3 (EP3 receptor) mRNA, cPLA2 mRNA, or COX2 mRNA, PTGES2 mRNA or PTGES3 mRNA (Fig 5a, 5b, 5c, 5d, 5g and 5h). There was a significant main effect of exercise for PGFS mRNA, which decreased in CTL (p<0.05) and ARA (p<0.05) Ex versus Non-Ex legs (Fig 5e). There was a significant main effect of exercise for PTGES mRNA, which increased in CTL (p<0.05) and ARA (p<0.01) Ex versus Non-Ex legs (Fig 5f). There was a significant main effect for exercise for IL-6 mRNA which increased following exercise (p<0.01) (p = 0.015), (Fig 5i and 5e). However, the high variation in IL-6 mRNA expression likely prevented within-group differences in the CTL and ARA groups when comparing Non-Ex to Ex muscles (p>0.10).

#### Myogenic-related mRNA Expression

**Fig 6:**
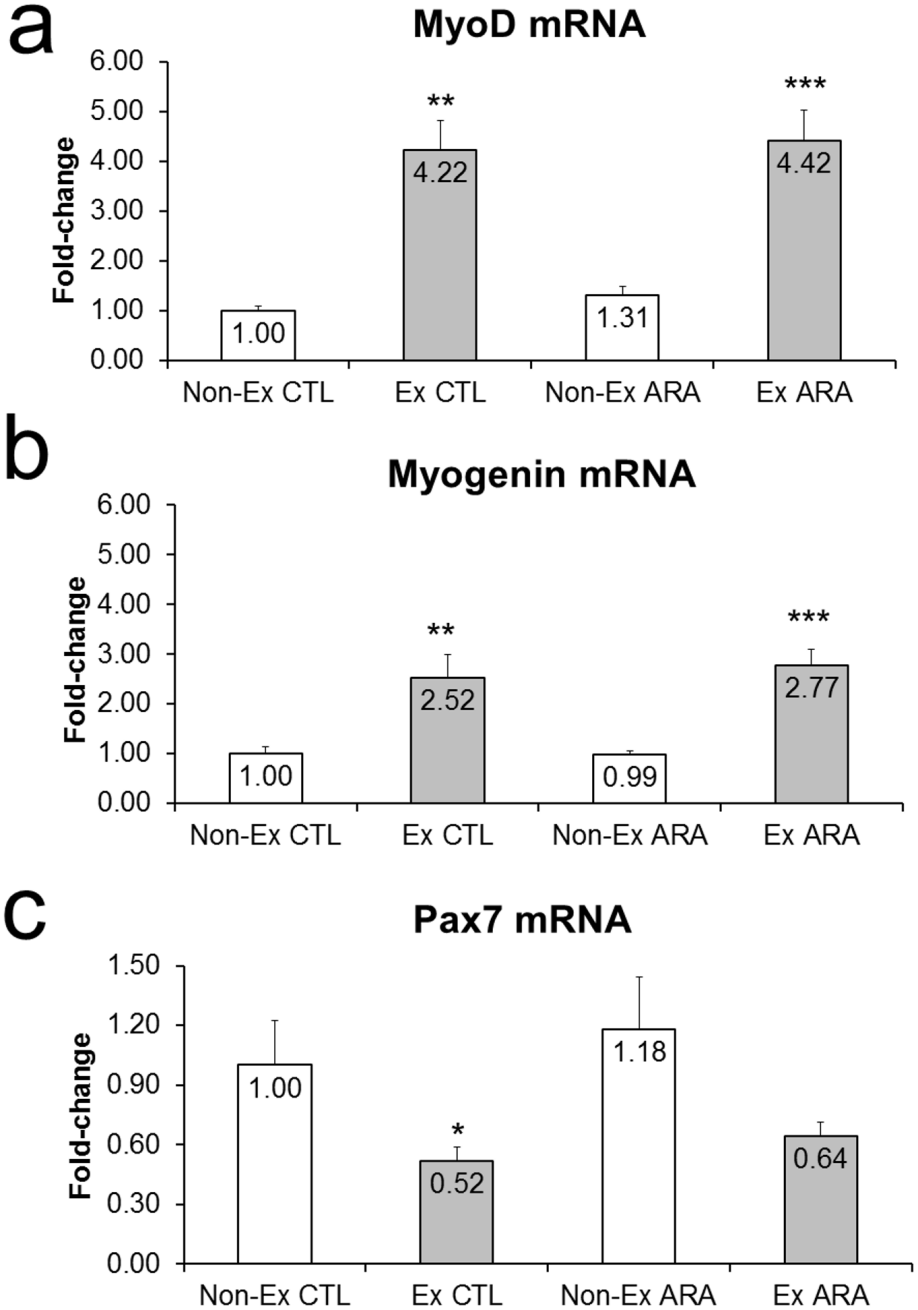
Fold-change values for MyoD mRNA (a), Myogenin mRNA (b), and Pax7 mRNA in non-exercised (open bars) and exercised (closed bars) rodents with (right) and without arachidonic acid supplementation.

The responses in myogenic-related mRNA expression following 8-days of ARA supplementation and acute exercise are presented in Fig 6. There were significant main effects for exercise for MyoD (increases, p<0.001), myogenin increases (p<0.01), and Pax7 mRNA (decreases, p = 0.0041).

## Discussion

The purpose of this study was two-fold: 1) to examine functional performance and body composition effects of 1.5g•d^-1^ of ARA supplementation in strength-trained individuals participating in a non-periodized, strength-training program; and 2) to examine if 8-days of ARA supplementation in rats enhanced post-exercise anabolic signaling mechanisms and muscle protein synthesis. We hypothesized that trained-individuals, undergoing ARA supplementation combined with strength training, would demonstrate greater functional performance and body composition changes, and that ARA supplementation would increase post-exercise anabolic signaling and protein synthesis in rat skeletal muscle. We partially confirmed our hypothesis as one of the main findings of the current study was that participants undergoing ARA supplementation combined with strength-training for 8 weeks significantly increased LBM and upper body strength compared to the placebo condition. However, following 8-days of ARA supplementation and acute strength exercise, ARA supplementation did not further enhance muscle protein synthesis up to 3 hours post-exercise and selected mRNAs in rodents compared to the control condition. Nevertheless, semi-chronic ARA supplementation appears to increase the phosphorylation (Ser9) of GSK-3β independent of high-frequency stimulation mimicking acute strength training. Semi-chronic ARA supplementation also resulted in decreased phosphorylation (Thr172) of AMPK-α following the acute strength training in rodents.

### ARA Supplementation and Strength-training Adaptations in Humans

In the current study, there was a time effect where both Ex ARA and Ex CTL groups significantly increased muscle thickness (p<0.0001). However, for LBM, there was a significant group by time interaction where post-hoc comparisons revealed that only the ARA group significantly increased LBM following 8 weeks of supplementation (i.e. 2.9%, p<0.0007). The proposed mechanism which ARA can positively increase muscle mass is associated with a previous study which demonstrated that, *in vitro*, ARA supplementation stimulates prostaglandins release and skeletal muscle hypertrophy via a COX-2 dependent pathway [9]. However, to date, there are conflicting results on the chronic effects of ARA supplementation and its anabolic mechanism in human skeletal muscle [17, 19, 25, 26]. Some chronic studies in older and younger populations demonstrated no interfering effects of COX inhibitors (which prevent ARA-derived prostaglandins formation) on skeletal muscle adaptations [17, 25, 26]. For instance, Trappe et al. (2011) reported when acetaminophen or ibuprofen was consumed during a strength-training regimen, they did not inhibit muscle hypertrophy or strength gains in older adults. The authors speculated that COX inhibition produced by the drugs could have a relatively stronger inhibitory effect on muscle protein breakdown than protein synthesis. Finally, despite our findings demonstrating a positive effect of ARA supplementation in LBM gains in strength-trained individuals, the current literature does not give rise to a proposed mechanism for these positive outcomes reported herein. Still, the effects of ARA supplementation on functional performance and body composition in trained subjects is sparse and our findings partially agree with previous data that investigated ARA supplementation in younger strength-trained males [19]. Specifically, Roberts et al. (2007) reported that 50 days of ARA supplementation with strength training improved lower body PP compared to a placebo group although changes in muscle mass and strength were similar between groups [19]. It should be noted that some differences exist between the aforementioned study and the present design. For one, Roberts et al. (2007) utilized a periodized strength-training program throughout the course of the study. In contrast, the training in the current study was intentionally stagnated (e.g. non-periodized regimen) in order to induce a training plateau in those strength-trained males. Another difference in the study design by Roberts et al. (2007) when compared to the present work is that participants were supplemented with 1g•d^-1^ of 40% ARA-containing oil as opposed to 1.5g•d^-1^ in the current study. This data may indicate a dose-response relationship where 1g•d^-1^ of ARA-containing oil may not provide ergogenic effects. This hypothesis is supported by the findings of Kelley et al. (1998) who demonstrated that the human consumption of 1.5g• d^-1^ of ARA significantly increased prostaglandin synthesis [27]. Finally, given that physically-active individuals possess less ARA and omega-6 fatty acids in muscle phospholipids due to increased turnover or demand [5–7], it may be suggested that higher daily doses of ARA (i.e., 1.5–2g•d^-1^ vs. 1g•d^-1^) may prove to be a more efficacious dose in strength-trained individuals.

Regarding the muscle performance data, interestingly, the current findings demonstrated that ARA supplementation did not induce better improvements in lower-body strength performance when compared to the PLA condition (17.8% vs 13.4%). Only the Ex ARA group demonstrated a significant increase in total-body strength (i.e. 95% CI_diff_ not crossing zero). In addition, there was a significant group by time interaction for upper-body strength (p<0.0001) where post-hoc comparisons revealed that only the Ex ARA group significantly increased 1RM bench-press (8.7% vs 3.8%, p<0.0001). Despite our findings suggesting a positive effect of ARA supplementation on muscle strength in strength-trained males, future studies will be needed to understand the role of ARA supplementation to induce positive muscle strength adaptations in the trained population.

The current findings, as well as prior one (Roberts et al., 2007), reported an improvement in PP followed by ARA supplementation. In the present study, there was a significant interaction (p<0.02) where post-hoc comparisons revealed that only the Ex ARA group significantly increased PP following 8 weeks of supplementation (10.7% vs. 3.7%, p<0.0001). Collectively, these findings suggest that ARA may function through an alternative pathway to increase neuromuscular efficiency. Literature has displayed the effects of ARA on pathways related to the mobilization of intracellular calcium [14, 28, 29]. While this appears to be initiated by PGF_2_ [30], there is also evidence that intracellular calcium mobilization is counterbalanced by PGE_2_ [31]. Still, it is plausible that transient increases in calcium may facilitate the release of neurotransmitters across the synaptic cleft and manifest in elevated power output. In fact, ARA has been demonstrated to enhance synaptic transmission in the hippocampus [32]. In this way, the effects of ARA on performance could be a more direct mechanism related to neurotransmission or contractile function of skeletal muscle (through alterations of intracellular calcium-release).

### ARA on Select Anabolic, Anti-catabolic Signaling Markers and Muscle Protein Synthesis Following Acute Exercise in Rodents

In order to address the effects of acute strength-training (i.e., semi-chronic) on molecular responses related to anabolic and catabolic signaling pathways and muscle protein synthesis following 8-days of ARA supplementation, we used a high-frequency electrical stimulation rat model to mimic a strength-training stimulus based on its efficacy to promote skeletal muscle hypertrophy in long-term interventions [21]. As previously mentioned, the metabolism of ARA in the 2-series prostaglandins was hypothesized to be the primary mechanism by which ARA would increase skeletal muscle hypertrophy. This contention is supported by findings from Trappe et al. (2001 & 2002) that demonstrated a correlation between prostaglandins and skeletal muscle fractional synthetic rate in humans post-exercise [16, 18].

Contrary to our hypothesis, molecular data collected from exercised rats supplemented with ARA did not present increases in selected mRNAs related to PGF_2α_ formation/signaling and/or muscle protein synthesis up to 3 hours post-exercise. Nevertheless, our data suggests ARA supplementation may be associated with hypertrophic properties through alternative pathways. For instance, there was a group effect increasing Ser-9 phosphorylation of GSK-3β in ARA versus CTL. Moreover, basal phosphorylation of GSK-3β was greater in ARA vs CTL (p<0.01). GSK-3β is a protein-serine kinase implicated in cell fate determination and differentiation. Moreover, GSK-3β is active in resting cells and inhibited when Ser9 phosphorylation occurs. This phosphorylation appears extremely important for initiating protein synthesis as GSK-3β is a known inhibitor of eIF2B, a rate-limiting enzyme in translation initiation [33]. Thus, while ARA supplementation did not enhance 3-h post-exercise muscle protein synthesis levels, interrogating the muscle protein synthesis response during later post-exercise time points may have yielded insightful information regarding whether ARA-induced increases in GSK-3β phosphorylation potentiated the anabolic response.

While interesting, it is difficult to determine the mechanism by which ARA supplementation may lead to increased phosphorylation of GSK-3β. On a basic level, supplementing with additional omega-6 fatty acids and their subsequent incorporation into membrane phospholipids may serve to improve membrane fluidity and insulin sensitivity [34, 35]. Nevertheless, future research is needed with regards to examining how ARA increases GSK-3β phosphorylation and/or if this event is related to a potentiation in post-exercise muscle protein synthesis rates beyond the 0–3 h post-exercise time window.

In addition, we reported a group effect for p-AMPK (Thr172), in which phosphorylation of AMPK only decreased in the exercising ARA group, but not in the control condition. The Thr172 phosphorylation of AMPK-α is a hallmark marker of decreased AMPK activity [36]. As a sensor of intracellular energy, activation in AMPK activity is typically associated with suppression in protein synthesis rate and inhibition of anabolic pathways including the Akt-mTOR pathway in rodents in acute fashion [37, 38]. Thus, again, these findings suggest a mechanistic link whereby ARA supplementation may potentiate post-exercise skeletal muscle hypertrophy beyond the immediate post-exercise window and warrants further investigation.

It is noteworthy to indicate that the aforementioned ARA-induced adaptations did not significantly affect muscle protein synthesis and selected intramuscular signaling markers investigated in this study. Thus, our findings did not accord with previous studies that demonstrated an inhibition on muscle protein synthesis rate in response to increase in the AMPK phosphorylation [38, 39]. To this end, studies implementing ARA supplementation in chronically trained humans or rodents will continue to unveil the mechanism by which ARA may, or may not, increase muscle mass or impact exercise stimuli.

## Conclusions

This study investigated the effects of ARA supplementation in trained males participating in an 8-week, non-periodized, strength-training program. Additionally, a separate rodent model was used to investigate the effects of high-frequency electrical stimulation to mimic a strength-training stimulus on acute post-exercise anabolic and catabolic signaling markers and muscle protein synthesis following 8-days of ARA supplementation. Our results suggest that strength-trained individuals can have a beneficial effect on LBM and muscle power supplementing with ARA. Indeed, our study has inherent limitations, first, we were not able to determine if ARA supplementation increased membrane phospholipids levels of ARA in both of our trials. Thus, it still remains to be determined whether ARA supplementation increases the ARA content in membrane phospholipids. Next, the rodent study was an 8-day supplementation protocol, which analyzed one post-exercise period (0–3 h). Future protocols examining different post-exercise periods following ARA supplementation are warranted. Notwithstanding, while ARA did not further enhance muscle protein synthesis up to 3 hours post-exercise, ARA supplementation did favorably alter the phosphorylation status of GSK-3β and AMPK. These signaling effects may potentiate an anabolic post-exercise environment beyond 3 hours. In addition, a considerable limitation of the translational studies is the ability of animal models to translate the complex mechanisms and adaptations reported in humans. Finally, further studies are warranted to demonstrate the anabolic role of ARA on training-induced adaptations.
